# Clustering of childhood acute leukemia in Finland: a nationwide register-based study

**DOI:** 10.1007/s10552-025-01998-1

**Published:** 2025-04-24

**Authors:** Julia Ventelä, Mia Korja, Anssi Auvinen, Olli Lohi, Atte Nikkilä

**Affiliations:** 1 Faculty of Medicine and Health Technology, Tampere University, Tampere, Finland; 2 Faculty of Social Sciences, Tampere University and Tampere University Hospital, Tampere, Finland; 3 Tampere Center for Child, Adolescent, Maternal Health Research and Tays Cancer Center, Tampere University and Tampere University Hospital, Tampere, Finland

**Keywords:** Acute leukemia, Clustering, Pediatric, Type 1 Diabetes, Epidemiology

## Abstract

**Purpose:**

Acute leukemia is the most common childhood malignancy, with suspected contributions from environmental factors and immune responses to common pathogens. A recent meta-analysis indicated possible spatiotemporal clustering, though the findings were hindered by data quality limitations. We investigated spatial and spatiotemporal clustering of childhood leukemia using advanced methods and complete residential histories.

**Methods:**

We included patients aged 0–17 years diagnosed in 1990–2019, using data from the Finnish Cancer Registry. A 1:3 age- and sex-matched case–control design was employed and residential history data with exact coordinates was collected. Clustering was evaluated using the Cuzick-Edwards test, Knox test, Kulldorff’s scan statistic, and Jacquez’s Q statistic.

**Results:**

The dataset included 1,626 childhood leukemia cases (median age 5.0 years, 54% male). The Knox test revealed no evidence of spatiotemporal clustering. However, the Cuzick-Edwards test revealed spatial clustering at diagnosis addresses for children under 1 year (OR 1.35, 95% CI 1.14–1.57). Further analysis with Jacquez’s Q test using complete residential histories identified significant spatiotemporal clustering in young children (ages 1.5–5.99 years) with acute lymphoblastic leukemia (ALL, *p* = 0.037). We also tested for co-incidence between leukemia and type 1 diabetes but found no clustering.

**Conclusion:**

Overall, we found limited evidence for clustering. In the subgroup analyses, significant spatiotemporal clustering in acute lymphoblastic leukemia cases among children aged 1.5–5.99 years was observed, coinciding with the peak incidence in early childhood. Previous research has shown that this age group has distinct genetic characteristics and may possess a unique etiology.

**Supplementary Information:**

The online version contains supplementary material available at 10.1007/s10552-025-01998-1.

## Background

Acute leukemia is the most common malignancy in children, yet its etiology remains largely unknown. Several somatic changes are required for the development of leukemia, and according to a prevailing hypothesis, the first often occurs during fetal development. One or several factors triggering secondary mutations are needed, but their significance is still poorly understood. [1, 2] These secondary mutations have been suggested to arise in connection with an abnormal immune reaction during childhood exposure to a delayed infection due to e.g., specific population mixing [3–5]. In addition to infections, other factors such as environmental or social factors could contribute to the disease etiology as well [6].

The first reports of epidemic-type clustering of leukemia were published in the 1960s [7]. Subsequent studies, and a recent meta-analysis, investigating the spatiotemporal clustering of childhood leukemia have produced mixed results, with some indicating moderate clustering [8, 9].

Type 1 diabetes is an autoimmune disease leading to the destruction of the insulin-producing β-cells in the pancreas. Despite distinct pathogeneses, evidence suggesting similar environmental exposures in the etiology of the two diseases has been reported [10]. Notably, both diseases exhibit higher incidence rates in developed countries, with Finland reporting one of the highest global incidences of type 1 diabetes, 56.8 per 100,000 person-years during 2000–2022 [11]. These parallels indicate potential shared environmental triggers, such as viral infections [10]. Previous studies examining the co-clustering of these diseases have found correlated incidence patterns and similar large-scale distributions [12–14].

The aim of this study was to further investigate spatial and spatiotemporal clustering of childhood acute leukemia and to investigate whether it shares spatiotemporal patterns with type 1 diabetes.

## Materials and methods

### Data

We identified childhood acute leukemia cases diagnosed in Finland from the Finnish Cancer Registry using International Classification of Diseases for Oncology, 3rd Edition (ICD-O-3) codes M9800–M9948. Diabetes cases were identified from the Social Insurance Institution of Finland (Kela) using special reimbursements for diabetes medication. Our inclusion criteria encompassed ages from 0 to 18 years and diagnosis between 1990 and 2019.

For each leukemia case, three sex- and age- matched controls were randomly sampled from the Population Information System of Finland. The age matching criterion was the same birth year. Each control was assigned a reference date corresponding to the age of their respective case at the time of his/her diagnosis. The conclusion of the exposure period was defined as the diagnosis date for cases and the reference date for the controls. Residential histories from birth to the diagnosis or reference date for the study participants were retrieved from the Digital and Population Data Services Agency (DVV). The data included the start and end dates of the residence periods, municipality, zip code, address and North and East coordinates in ETRS-TM35FIN.

The residential histories were restricted to addresses between birth and the date of leukemia diagnosis or the reference date for controls, resulting in 14,223 residencies. The residential periods lacking start or end dates were removed. Among leukemia cases, 5.1% (*n* = 83) lacked coordinates for some of their residential periods, comparable to 5.9% (*n* = 286) of controls.

**Fig. 1:**
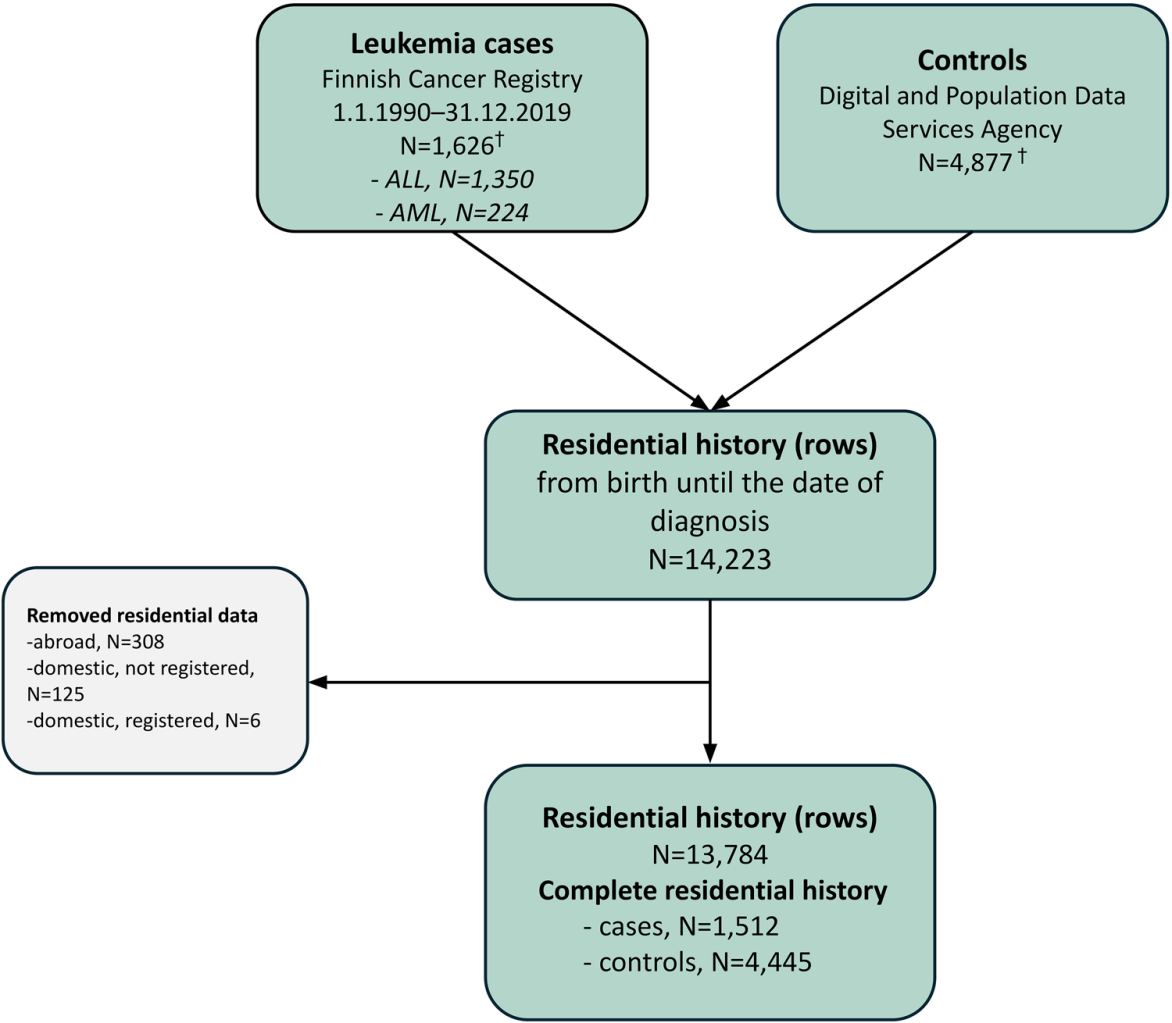
Flowchart of the study population and their residential data. ^†^Study cohort after excluding duplicate entries, along with their corresponding controls

Consequently, we excluded 439 instances of residential data: 308 periods abroad, 125 domestic periods not registered in the Population Information System, and 6 domestic periods registered in the Population Information but lacking documentation. This process yielded a dataset of 13,784 rows of residential data, with 3,426 rows allocated to 1,617 cases and 10,358 to 4,865 controls (Fig. 1). For 1,512 cases (93.0%) and 4,445 controls (91.1%), the residential history was complete with no missing data.

### Statistical analysis

Statistical analyses were conducted using R (v. 4.0.5, R Core Team, 2021, Vienna), Spacestat (BioMedware Inc., Ann Arbor, MI, v. 4.0.21), and SaTScan (v. 10.1.2) [15, 16]. All the analyses were performed in a secure remote environment provided by the Finnish Social and Health Data Permit Authority, Findata.

The analyses were conducted using four distinct approaches: full residential histories, the addresses at the time of diagnosis, the addresses one year preceding the diagnosis or the addresses at birth. A significance threshold of *p* < 0.05 was applied to determine statistically significant clustering, while 0.05 < *p* < 0.1 suggested possible clustering. We used Benjamini-Hochberg (BH) method to correct for multiple testing [17].

### Cuzick-Edwards test

The Cuzick-Edwards test evaluates the number and proportion of cases within the *k* = 5 and *k* = 15 nearest neighbors and relies solely on spatial analysis, utilizing coordinates of both cases and controls [18]. Spatial clustering was analyzed using both five and fifteen nearest neighbors to assess whether the results remained consistent as the number of nearest neighbors increased.

In our study, we adapted this test also to address spatial co-clustering of acute leukemia and diabetes, a method originally utilized in a Danish case–control study investigating co-clustering of the two diseases in Denmark [14]. Shortly, individuals with leukemia were considered as cases and type 1 diabetes cases served as controls. Rejecting the null hypothesis with a smaller test statistic than expected indicates leukemia cases exhibiting an excess of diabetes cases nearby. Additionally, we conducted the test also treating diabetes cases as cases and leukemia cases as their controls.

### Knox test

We utilized the Knox test to examine the spatiotemporal clustering patterns of cases with predefined thresholds: distance (0.25, 0.5, 1, 5, 10 km) and time (2, 6, 12, 18, 24 months) [19]. This test was implemented using the knox() function from the surveillance package (version 1. 20.0) in the R programming environment, with 10,000 iterations for leukemia cases, adjusted according to dataset size and computational capacity. To assess the statistical significance of the observed clustering patterns, Monte Carlo permutation tests were employed to derive *p*-values.

### Kulldorff’s space–time scan statistic

Kulldorff’s space–time scan statistic, implemented using the SaTScan program, was utilized to investigate spatiotemporal clustering [20, 21]. For each location, a cylindrical scanning window was used, with the base representing the spatial dimension and the height passing through time. A series of ellipses was constructed to encompass neighboring areas within their defined boundaries.

The parameters were configured to allow for a maximum spatial cluster size of up to 25% of the population at risk within ellipses with a 10 km radius. Due to excessively long computing times, case accrual was aggregated into three-month intervals. For the maximum temporal cluster size, the default settings were applied, with the upper limit set to 50% of the study period and the lower limit set to one month. However, due to data aggregation, the actual minimum temporal cluster size was three months. A Bernoulli probabilistic model, comparing cases and controls as binary variables, was utilized. To assess statistical significance, *p*-values were derived from Monte Carlo simulations, consisting of 999 replications.

### Jacquez’s Q statistic

We employed Jacquez’s Q statistic, utilizing the commercial Spacestat program, developed by BioMedware, to analyze spatiotemporal clustering based on complete residential histories [22]. To determine the optimal number of nearest neighbors (*k*) for the analysis, following the recommendation in the Spacestat Software documentation, we tested multiple values (5, 10, 15), evaluating the *p*-value of the global Q statistic for each. We selected *k* = 5 based for all cases and controls and *k* = 15 based for the largest subgroup ALL. This statistical method incorporates both spatial and temporal dimensions, providing insights into the likelihood of individuals belonging to clusters at various times and locations. The statistic is recalculated each time a study participant moves, allowing for a dynamic understanding of clustering patterns over an individual’s study period from birth to diagnosis/reference date.

To assess the statistical significance of the Q-statistics, we conducted Monte Carlo simulations with 599–999 iterations. Jacquez’s Q statistic yields four main indicators: the global Q, Q_i_, Q_t_, and Q_it_. The global Q statistic represents the average clustering across all individuals over time, while Q_i_ evaluates clustering around individual cases or controls throughout their lifespan. Q_t_ assesses global clustering at a specific time point, and Q_it_ examines clustering around an individual at a given time. Particularly interesting are the Q_it_ values whose cases have significant Q_i_ values. This combination indicates specific locations and times where cases with significant lifetime clustering are part of geographically localized clusters.

### Subgroups

The data were stratified by sex, age, and type of leukemia (acute lymphoblastic leukemia and acute myeloid leukemia). Due to the low number of B-cell (*n* = 226) and T-cell (*n* = 70) ALL, we could not include those more specific subtypes for subgroup analyses. The age groups considered for the leukemia dataset were derived a priori from its incidence distribution: 0–0.99, 1–9.99 and 10–17.99 years. Additionally, for ALL cases, the incidence peak age group 1.5–5.99 years was further evaluated. The aforementioned statistical analyses were conducted independently for each subgroup.

Additionally, the Knox test was performed for two distinct scenarios: cases with only a single residence prior to diagnosis date and for varying time periods categorized by the diagnosis date 1990–1999, 2000–2009, 2010–2019). This approach allowed for a comprehensive exploration of spatiotemporal clustering patterns across different demographic and temporal strata.

## Results

### Study population

In total, 1,762 leukemia cases were identified from the Finnish Cancer Registry. After excluding duplicates, we retained a cohort of 1,626 leukemia cases along with 4,877 controls. The median age at leukemia diagnosis was 4.97 years (IQR 2.85–10.33). The age distribution showed an expected peak incidence of leukemia within the age range of 1.5 to 5.99 years old. Among the leukemia cases, a slight majority were male, constituting 53.9% (*n* = 876). As anticipated, ALL was the predominant type of leukemia, accounting for 83.0% (*n* = 1,350) of all leukemia cases, with a median age of 4.86 years (IQR 2.98–9.53) (Table 1).

**Table 1:** Characteristics of the study population

	Cases, *n* (%)	Controls, *n* (%)
Total	1,626	4,877
Sex		
Female	750 (46.1)	2,250 (46.1)
Male	876 (53.9)	2,627 (53.9)
Age		
0–0.99 years	78 (4.8)	234 (4.8)
1–9.99 years	1,127 (69.3)	3,380 (69.3)
10–17.99 years	421 (25.9)	1,263 (25.9)
Type of leukemia		
ALL	1,350 (83.0)	4,049 (83.0)
ALL, 1.5–5.99 years	732 (45.0)	2,196 (45.0)
AML	224 (13.8)	672 (13.8)
Others	52 (3.2)	156 (3.2)
Residential history information		
Complete	1,512 (93.0)	4,445 (91.1)
Residence at birth	1,577 (97.0)	4,701 (96.4)
Residence one year prior to diagnosis	1,600 (98.4)	4,816 (98.7)
Residence at diagnosis	1,612 (99.1)	4,859 (99.6)

*ALL* Acute lymphoblastic leukemia, *AML* Acute myeloid leukemia

There was no significant difference between leukemia cases and controls regarding the number of residencies before the reference date (mean for leukemia cases 2.12, and mean for controls 2.13; *p* = 0.618, Wilcoxon rank sum test).

### Leukemia clustering

Leukemia clustering was assessed using various statistical methods. Initially, we applied the Cuzick-Edwards test, which detects solely spatial clustering by analyzing the proportion of cases among the k nearest neighbors for all instances. Using the five nearest neighbors, we examined clustering at three time points: diagnosis date, one year prior to diagnosis and birth date. No evidence of clustering was observed among all leukemia cases (Table S1). However, in subgroup analyses, clustering at the time of diagnosis was detected among children diagnosed before the age of one year (Obs/Exp = 1.35, 95% CI 1.14–1.57, *p* = 0.027) (Table S1). Increasing the number of nearest neighbors evaluated to fifteen did not yield significant results (Table S2).

For evaluation of spatiotemporal clustering, we utilized the Knox test, which quantifies, using prespecified thresholds, whether two diagnoses being close in time increases the probability of being close also in distance. Overall, subsequent analyses considering different types of leukemia or various time periods did not yield statistically significant spatiotemporal clustering patterns (Tables S3 and S4).

Kulldorff’s space–time scan statistic, which identifies clusters by scanning the study area with varying-sized windows to detect higher-than-expected case densities, did not identify any significant clusters in the analysis of the entire study population. Likewise, the subgroup analyses revealed no significant clustering, as all detected clusters detected had a *p*-value greater than 0.1 (Data not shown).

**Fig. 2:**
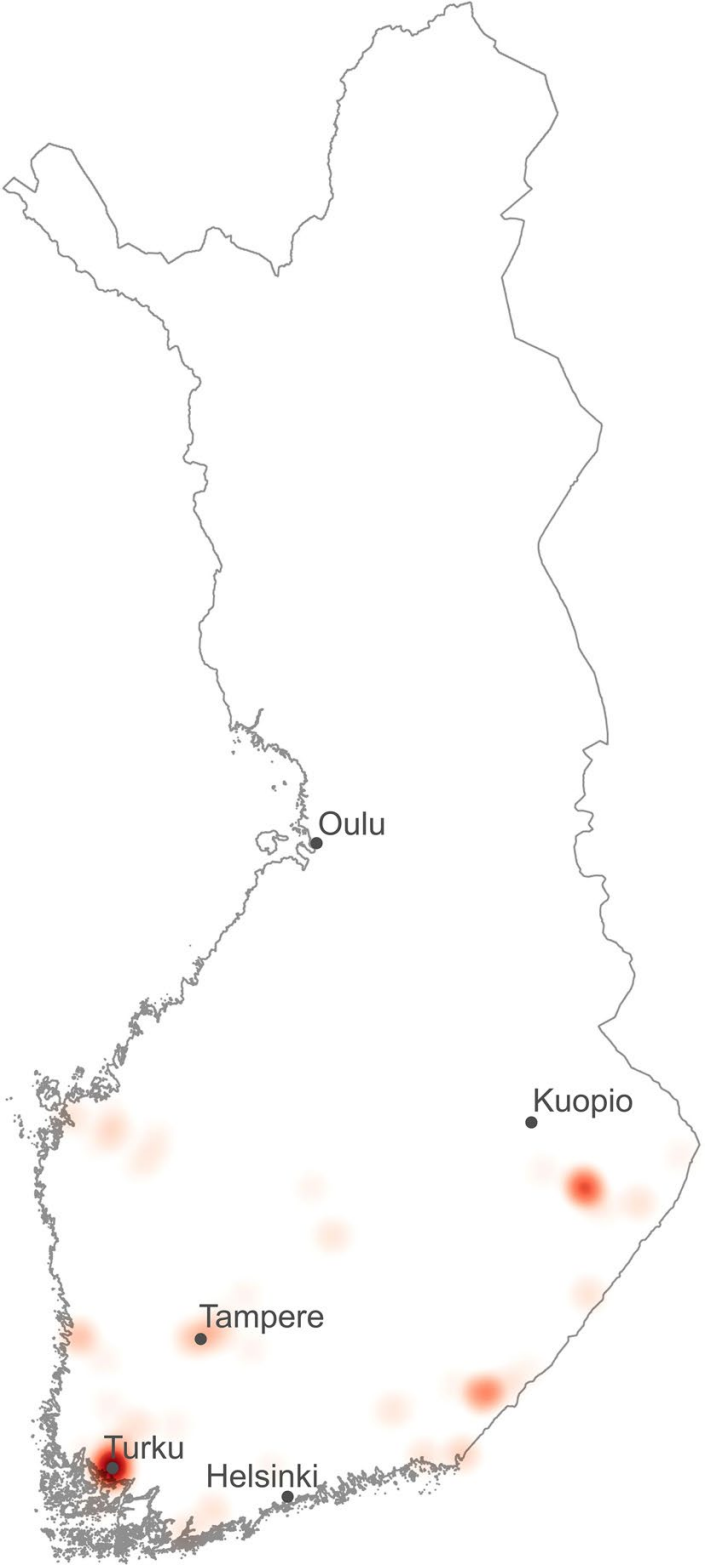
Heat map showing the locations of the identified significant Q_it_ values (Jacquez’s Q) among the subgroup of ALL 1.5–5.99 years old summarized from years 1972–2019

Method that uses the entire residential histories of both leukemia cases and their controls, Jacquez’s Q statistic, identified significant local clustering among the fifteen nearest neighbors for the subgroup comprising ALL cases aged 1.5–5.99 years. The results for this ALL subgroup, corresponding to the peak age range for the disease, indicated clustering around cases throughout their lifespan (Q_i_, *n* = 60, *p* = 0.008). Furthermore, significant Q_it_ statistics (*n* = 96, *p* = 0.037) were observed, indicating clustering at specific time points within this subgroup. To illustrate the locations of these significant clusters, we plotted the study subjects’ locations on a map of Finland, summarized from years 1990–2019 (Fig. 2). The significant clusters were disproportionately located in southwestern Finland, within a 50 km radius of the city of Turku, accounting for 24% (*n* = 23) of the significant Q_it_ values. In comparison, this region encompasses approximately 2.3% of Finland’s total land area and contains 5.5% of the country’s population. The presence of significant Q_it_ values that were also part of significant Q_i_ clusters (*n* = 74, *p* = 0.011) underscored the statistical weight of these findings. However, the global Q statistic was not significant (*p* = 0.14), and no significant temporal patterns were identified. Reducing the number of nearest neighbors analyzed to five yielded significant results only for the female subgroup, based on the number of significant Q_it_ values that were also part of significant Q_t_ values (*n* = 34, *p* = 0.019) as well as those Q_it_ values included in both significant Qi and Qt values (*n* = 24, *p* = 0.015). Similar analyses with *k* = 5 or *k* = 15 for other subgroups yielded no statistically significant results.

### Co-clustering of leukemia and type I diabetes

Given the potential links between childhood acute leukemia and type 1 diabetes, with shared environmental triggers and abnormal immune responses suggested by recent studies, we examined the spatial co-clustering of these diseases. To this end, we used a cohort of 16,307 cases diagnosed with type 1 diabetes and a dataset comprising 38,625 rows of residential data (Ventelä et al., manuscript in preparation). This analysis employed a modified version of the Cuzick-Edwards test, comparing 1,596 leukemia cases to 16,279 diabetes cases treated as controls. To account for the temporal dimension, we assessed potential clustering at three distinct time points: the date of diagnosis, one year prior to diagnosis, and birth date. The co-clustering of diseases was analyzed using both five and fifteen nearest neighbors to assess whether the results remained consistent as the number of nearest neighbors increased. The results suggested no evidence of co-clustering of the diseases (Tables S5 and S6).

We compiled summaries of the cluster analysis results by method and place of residence at three specific time points (Table 2).

**Table 2:**
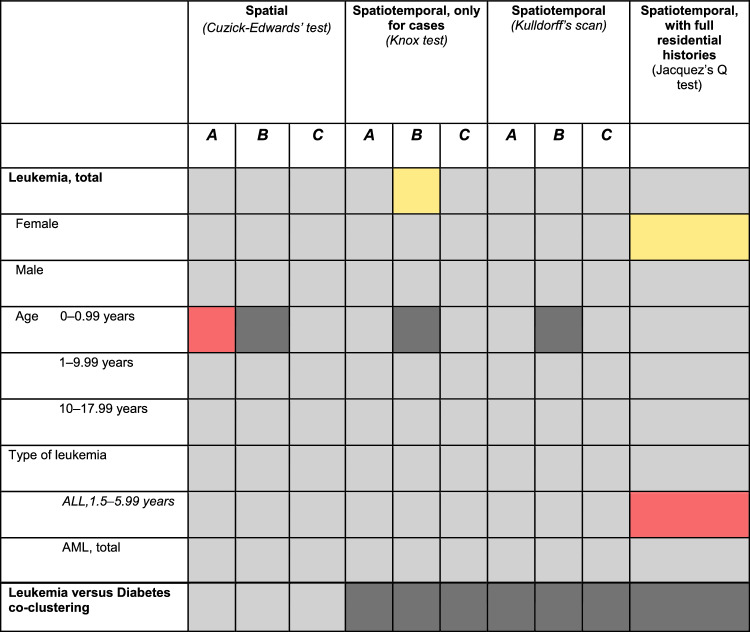
Summary of cluster analysis results by method and place of residence

Residence timing categories: **A** = At the time of diagnosis; **B** = One year prior to diagnosis; **C** = At birth

Color codes: Red = Significant clustering, *p* < 0.05, Yellow = Suggestive of clustering, 0.05 < *p* < 0.1, Light grey = No clustering, Dark grey = Not analyzed

*ALL* Acute lymphoblastic leukemia, *AML* Acute myeloid leukemia

## Discussion

This study investigated the spatiotemporal clustering of childhood leukemia and its potential spatial co-occurrence with type 1 diabetes in Finland. We employed a combination of established methods to analyze residential histories spanning birth to diagnosis (or reference date for controls). Overall, considering leukemia independently and in parallel with type 1 diabetes, we found limited evidence for clustering. In the subgroup analyses, our findings revealed significantly elevated spatiotemporal clustering for ALL in young children (1.5–5.99 years), coinciding with the peak incidence age in this cohort. This aligns with the known age distribution of ALL subtypes, particularly ETV6-RUNX1 and high hyperdiploidy, which are commonly prevalent in early childhood ALL [23]. A prior Swiss study investigating childhood leukemia clustering and potential clinical characteristics associated with it, reported a higher prevalence of the ETV6-RUNX1 gene translocation among leukemia cases born in close spatial and temporal proximity [24].

Employing the Cuzick-Edwards method, we identified statistically significant spatial clustering of leukemia cases diagnosed in infants under one year old. This suggests that these cases are concentrated in specific geographic areas, pointing to potential exposure to persistent risk factors over time, such as emissions from infrastructure. To our knowledge, prior evidence of spatial clustering in leukemia cases diagnosed in children under one year old is limited. However, previous studies have reported an increased risk of leukemia in children under five years old residing near highways and nuclear power plants [25–27].

A previous pooled analysis using the Knox test, summarizing prior research on spatiotemporal clustering of leukemia in individuals under 15 years old, also indicated borderline results (Obs/Exp = 1.01, *p* = 0.054). However, this analysis demonstrated statistically significant clustering specifically within the youngest age group, under 5 years old [8]. This aligns with our result of significant clustering among the ALL subgroup of young children aged 1.5–5.99 years which was located disproportionately in southwestern Finland. These findings support the hypothesis that exposure to infections, particularly those occurring in mini-epidemics during the peak incidence of ALL, may be a key etiological factor in childhood leukemia.

Our analysis using the Cuzick-Edwards test did not yield significant evidence for spatial co-clustering of type 1 diabetes and acute childhood leukemia. Only a few previous studies have explored the co-clustering of childhood acute leukemia and type 1 diabetes. The first suggestion of shared spatiotemporal clustering of the diseases emerged in the early 2000s when a UK study observed a positive spatial correlation between the diseases and a further extension of the study considering the temporal variation supported it: a positive correlation of at least 0.7 between diseases was observed across all time periods (from 1978 to 2003) [12, 13]. Also, a previous Danish case–control study identified co-clustering with the Cuzick-Edwards method for the ALL age group of 2–6 years old with type 1 diabetes, at both the place of diagnosis and when considering residential mobility [14].

This study leverages high-quality data from well-established Finnish registries, encompassing a large population of roughly 1.1 million children over three decades with full residential histories. A key strength lies in employing a diverse array of analytical methods and conducting comprehensive data analysis looking at all time points of interest with precise geographic coordinates. To analyze clustering with full residential histories, we utilized Jacquez’s Q-statistics, thereby providing a more nuanced understanding of potential spatiotemporal clustering compared to methods focused on a single time point.

The study has several limitations. The clustering analysis methods depend on user-defined thresholds, which may influence the sensitivity in detecting subtle clustering patterns. Conversely, the potentially low specificity of the models could lead to false positive results. Another significant constraint of the analyses was the limited computational power, as multicore processing was not possible with the software available. Additionally, the dataset size, while adequate for this study, is relatively small on a global scale. A further limitation was the limited classification of immunophenotype, which prevented reliable subgroup analyses. The most common morphological code in our dataset was “precursor cell lymphoblastic leukemia, NOS,” which limited accurate classification of B-ALL and T-ALL subtypes.

Our findings indicate spatiotemporal clustering of ALL among young children, which is especially interesting as this subgroup has been hypothesized to have a distinct etiology. Subsequent studies could delve deeper into these identified clusters to identify common factors, with a particular focus on southwestern Finland. The evidence for co-clustering of leukemia and type 1 diabetes remains inconclusive. Future investigations should aim to validate our finding on the clustering of ALL in its incidence peak, stratify these analyses by specific ALL subtypes and to look for explanations for these clusters.

## Supplementary Information

Below is the link to the electronic supplementary material.

Supplementary file1 (DOCX 23 KB)

Supplementary file2 (DOCX 23 KB)

Supplementary file3 (DOCX 23 KB)

Supplementary file4 (DOCX 58 KB)

Supplementary file5 (DOCX 22 KB)

Supplementary file6 (DOCX 23 KB)

## Data Availability

No datasets were generated or analysed during the current study.
