# Supplementary material for: Clustering of childhood acute leukemia in Finland: a nationwide register-based study
Source: Cancer Causes Control. 2025 Apr 24;36(9):957–64. doi: 10.1007/s10552-025-01998-1 (PMC12380987; doi:10.1007/s10552-025-01998-1)
Supplement: Supplementary file 1 — Supplementary file1 (DOCX 23 KB) [file 10552_2025_1998_MOESM1_ESM.docx]

| **Table S1.** Results of the Cuzick-Edwards’ test (k=5) for leukemia cases and their controls based on place of residence with three residence timing categories. |
| --- |
| \| **At the time of diagnosis** \| **Subgroup** \| **Obs** \| **Exp** \| **Obs/Exp** \| **95% CI** \| **p-value^†^** \| \| --- \| --- \| --- \| --- \| --- \| --- \| --- \| \| **Leukemia** \| All \| 2,002 \| 2,007 \| 1.00 \| 0.95–1.04 \| 0.92 \| \| Sex \| Female \| 997 \| 924 \| 1.08 \| 1.01–1.15 \| 0.17 \| \|  \| Male \| 1,009 \| 1,082 \| 0.93 \| 0.87–1.00 \| 0.17 \| \| Age, years \| 0–0.99 \| 131 \| 97 \| **1.35** \| **1.14–1.57** \| **0.027** \| \|  \| 1–9.99 \| 1,412 \| 1,402 \| 1.01 \| 0.95–1.06 \| 0.85 \| \|  \| 10–17.99 \| 484 \| 520 \| 0.93 \| 0.84–1.02 \| 0.37 \| \| Leukemia subtype, years \| ALL \| 1,649 \| 1,680 \| 0.98 \| 0.93–1.03 \| 0.65 \| \|  \| ALL, 1.5–5.99 \| 920 \| 912 \| 1.01 \| 0.94–1.08 \| 0.85 \| \|  \| AML \| 285 \| 274 \| 1.04 \| 0.91–1.17 \| 0.69 \| |
| \| **One year prior to diagnosis** \| **Subgroup** \| **Obs** \| **Exp** \| **Obs/Exp** \| **95% CI** \| **p-value^†^** \| \| --- \| --- \| --- \| --- \| --- \| --- \| --- \| \| **Leukemia** \| All \| 1,955 \| 1,994 \| 0.98 \| 0.93–1.03 \| 0.58 \| \| Sex \| Female \| 974 \| 924 \| 1.05 \| 0.98–1.12 \| 0.37 \| \|  \| Male \| 987 \| 1,069 \| 0.92 \| 0.86–0.99 \| 0.17 \| \| Age, years \| 0–0.99 \| NA \| NA \| NA \| NA \| NA \| \|  \| 1–9.99 \| 1,370 \| 1,403 \| 0.98 \| 0.92–1.03 \| 0.58 \| \|  \| 10–17.99 \| 490 \| 517 \| 0.95 \| 0.86–1.04 \| 0.49 \| \| Leukemia subtype, years \| ALL \| 1,629 \| 1,682 \| 0.97 \| 0.92–1.02 \| 0.46 \| \|  \| ALL, 1.5–5.99 \| 903 \| 917 \| 0.98 \| 0.92–1.05 \| 0.81 \| \|  \| AML \| 285 \| 269 \| 1.06 \| 0.93–1.19 \| 0.58 \| |
|  |
| \| **At birth** \| **Subgroup** \| **Obs** \| **Exp** \| **Obs/Exp** \| **95% CI** \| **p-value^†^** \| \| --- \| --- \| --- \| --- \| --- \| --- \| --- \| \| **Leukemia** \| All \| 2,075 \| 1,980 \| 1.05 \| 1.00–1.10 \| 0.17 \| \| Sex \| Female \| 921 \| 910 \| 1.01 \| 0.94–1.08 \| 0.85 \| \|  \| Male \| 1,119 \| 1,068 \| 1.05 \| 0.98–1.11 \| 0.37 \| \| Age, years \| 0–0.99 \| 118 \| 97 \| 1.22 \| 1.01–1.43 \| 0.17 \| \|  \| 1–9.99 \| 1,464 \| 1,401 \| 1.04 \| 0.99–1.10 \| 0.37 \| \|  \| 10–17.99 \| 530 \| 527 \| 1.01 \| 0.92–1.10 \| 0.92 \| \| Leukemia subtype, years \| ALL \| 1,723 \| 1,682 \| 1.02 \| 0.97–1.08 \| 0.58 \| \|  \| ALL (1.5–5.99 years old) \| 958 \| 915 \| 1.05 \| 0.98–1.12 \| 0.41 \| \|  \| AML \| 297 \| 278 \| 1.07 \| 0.95–1.20 \| 0.46 \|  \| \| *^†^Benjamini–Hochberg adjusted p-value*  *Bold type: p-value < 0.05.*  *Abbreviations: Obs, observed; Exp, expected; CI, confidence interval; ALL, Acute lymphoblastic leukemia; AML, Acute myeloid leukemia; NA, Not applicable* \| \| --- \| \| \| --- \| --- \| |
