# Supplementary material for: Clustering of childhood acute leukemia in Finland: a nationwide register-based study
Source: Cancer Causes Control. 2025 Apr 24;36(9):957–64. doi: 10.1007/s10552-025-01998-1 (PMC12380987; doi:10.1007/s10552-025-01998-1)
Supplement: Supplementary file 2 — Supplementary file2 (DOCX 23 KB) [file 10552_2025_1998_MOESM2_ESM.docx]

| **Table S2.** Results of the Cuzick-Edwards’ test (k=15) for leukemia cases and their controls based on place of residence with three residence timing categories. |
| --- |
| \| **At the time of diagnosis** \| **Subgroup** \| **Obs** \| **Exp** \| **Obs/Exp** \| **95% CI** \| **p-value^†^** \| \| --- \| --- \| --- \| --- \| --- \| --- \| --- \| \| **Leukemia** \| All \| 6,008 \| 6,021 \| 1.00 \| 0.97–1.03 \| 0.97 \| \| Sex \| Female \| 2,920 \| 2,771 \| 1.05 \| 1.01–1.10 \| 0.31 \| \|  \| Male \| 3,181 \| 3,247 \| 0.98 \| 0.94–1.02 \| 0.97 \| \| Age, years \| 0–0.99 \| 311 \| 290 \| 1.07 \| 0.94–1.20 \| 0.97 \| \|  \| 1–9.99 \| 4,202 \| 4,205 \| 1.00 \| 0.96–1.03 \| 0.97 \| \|  \| 10–17.99 \| 1,570 \| 1,560 \| 1.01 \| 0.95–1.06 \| 0.97 \| \| Leukemia subtype, years \| ALL \| 4,980 \| 5,039 \| 0.99 \| 0.96–1.05 \| 0.97 \| \|  \| ALL, 1.5–5.99 \| 2,746 \| 2,735 \| 1.00 \| 0.96–1.05 \| 0.97 \| \|  \| AML \| 850 \| 822 \| 1.03 \| 0.95–1.11 \| 0.97 \| |
| \| **One year prior to diagnosis** \| **Subgroup** \| **Obs** \| **Exp** \| **Obs/Exp** \| **95% CI** \| **p-value^†^** \| \| --- \| --- \| --- \| --- \| --- \| --- \| --- \| \| **Leukemia** \| All \| 5,976 \| 5.982 \| 1.00 \| 0.97–1.03 \| 0.97 \| \| Sex \| Female \| 2,912 \| 2,773 \| 1.05 \| 1.01–1.09 \| 0.31 \| \|  \| Male \| 3,177 \| 3,206 \| 0.99 \| 0.95–1.03 \| 0.97 \| \| Age, years \| 0–0.99 \| NA \| NA \| NA \| NA \| NA \| \|  \| 1–9.99 \| 4,158 \| 4,208 \| 0.99 \| 0.95–1.02 \| 0.97 \| \|  \| 10–17.99 \| 1,549 \| 1,551 \| 1.00 \| 0.94–1.06 \| 0.97 \| \| Leukemia subtype, years \| ALL \| 4,915 \| 5,046 \| 0.97 \| 0.94–1.01 \| 0.95 \| \|  \| ALL, 1.5–5.99 \| 2,763 \| 2,751 \| 1.00 \| 0.96–1.05 \| 0.97 \| \|  \| AML \| 828 \| 808 \| 1.02 \| 0.94–1.11 \| 0.97 \| |
|  |
| \| **At birth** \| **Subgroup** \| **Obs** \| **Exp** \| **Obs/Exp** \| **95% CI** \| **p-value^†^** \| \| --- \| --- \| --- \| --- \| --- \| --- \| --- \| \| **Leukemia** \| All \| 6,002 \| 5,939 \| 1.01 \| 0.98–1.04 \| 0.97 \| \| Sex \| Female \| 2,778 \| 2,731 \| 1.02 \| 0.97–1.06 \| 0.97 \| \|  \| Male \| 3,257 \| 3,205 \| 1.02 \| 0.98–1.06 \| 0.97 \| \| Age, years \| 0–0.99 \| 304 \| 290 \| 1.05 \| 0.92–1.18 \| 0.97 \| \|  \| 1–9.99 \| 4,231 \| 4,204 \| 1.01 \| 0.97–1.04 \| 0.97 \| \|  \| 10–17.99 \| 1,589 \| 1,580 \| 1.00 \| 0.95–1.06 \| 0.97 \| \| Leukemia subtype, years \| ALL \| 5,057 \| 5,046 \| 1.00 \| 0.97–1.03 \| 0.97 \| \|  \| ALL (1.5–5.99 years old) \| 2,779 \| 2,744 \| 1.01 \| 0.97–1.06 \| 0.97 \| \|  \| AML \| 841 \| 830 \| 1.01 \| 0.93–1.09 \| 0.97 \|  \| \| *^†^Benjamini–Hochberg adjusted p-value*  *Bold type: p-value < 0.05.*  *Abbreviations: Obs, observed; Exp, expected; CI, confidence interval; ALL, Acute lymphoblastic leukemia; AML, Acute myeloid leukemia; NA, Not applicable* \| \| --- \| \| \| --- \| --- \| |
