# Supplementary material for: Clustering of childhood acute leukemia in Finland: a nationwide register-based study
Source: Cancer Causes Control. 2025 Apr 24;36(9):957–64. doi: 10.1007/s10552-025-01998-1 (PMC12380987; doi:10.1007/s10552-025-01998-1)
Supplement: Supplementary file 3 — Supplementary file3 (DOCX 23 KB) [file 10552_2025_1998_MOESM3_ESM.docx]

**Table S3.** The Knox test results (Benjamini-Hochberg adjusted p-values) for leukemia cases in distance and time with three residence timing categories.

1. Residence at diagnosis

| time (months)  distance (m) | 2 | 6 | 12 | 18 | 24 |
| --- | --- | --- | --- | --- | --- |
| 250 | 0.75 | 0.41 | 0.20 | 0.20 | 0.20 |
| 500 | 0.86 | 0.65 | 0.41 | 0.20 | 0.28 |
| 1,000 | 0.83 | 0.41 | 0.20 | 0.20 | 0.20 |
| 5,000 | 0.41 | 0.41 | 0.43 | 0.74 | 0.86 |
| 10,000 | 0.74 | 0.43 | 0.41 | 0.41 | 0.43 |

1. Residence one year prior to diagnosis

| time (months)  distance (m) | 2 | 6 | 12 | 18 | 24 |
| --- | --- | --- | --- | --- | --- |
| 250 | 0.81 | 0.33 | 0.13 | 0.059 | 0.10 |
| 500 | 0.66 | 0.22 | 0.073 | 0.073 | 0.13 |
| 1,000 | 0.81 | 0.33 | 0.059 | 0.059 | 0.059 |
| 5,000 | 0.81 | 0.81 | 0.81 | 0.81 | 0.81 |
| 10,000 | 0.96 | 0.81 | 0.81 | 0.81 | 0.81 |

1. Residence at birth

| time (months)  distance (m) | 2 | 6 | 12 | 18 | 24 |
| --- | --- | --- | --- | --- | --- |
| 250 | 0.79 | 0.94 | 0.94 | 0.94 | 0.98 |
| 500 | 0.74 | 0.73 | 0.73 | 0.74 | 0.74 |
| 1,000 | 0.73 | 0.73 | 0.74 | 0.74 | 0.79 |
| 5,000 | 0.98 | 0.98 | 0.94 | 0.74 | 0.73 |
| 10,000 | 0.94 | 0.94 | 0.94 | 0.74 | 0.73 |

*Color codes: Yellow = Suggestive of clustering, 0.05 < p < 0.1, Light grey = No clustering*
