# Supplementary material for: Clustering of childhood acute leukemia in Finland: a nationwide register-based study
Source: Cancer Causes Control. 2025 Apr 24;36(9):957–64. doi: 10.1007/s10552-025-01998-1 (PMC12380987; doi:10.1007/s10552-025-01998-1)
Supplement: Supplementary file 4 — Supplementary file4 (DOCX 58 KB) [file 10552_2025_1998_MOESM4_ESM.docx]

**Table S4.** The Knox test results (Benjamini-Hochberg adjusted p-values) for leukemia cases in distance (rows, meters) and time (columns, months) with three residence timing categories.

1. female (date of diagnosis, one year prior to diagnosis, date of birth)

|  | 2 | 6 | 12 | 18 | 24 |
| --- | --- | --- | --- | --- | --- |
| 250 | 1 | 1 | 1 | 1 | 1 |
| 500 | 1 | 1 | 1 | 1 | 1 |
| 1,000 | 1 | 1 | 1 | 1 | 1 |
| 5,000 | 1 | 1 | 1 | 1 | 1 |
| 10,000 | 1 | 1 | 1 | 1 | 1 |

|  | 2 | 6 | 12 | 18 | 24 |
| --- | --- | --- | --- | --- | --- |
| 250 | 1 | 1 | 1 | 1 | 1 |
| 500 | 1 | 1 | 1 | 1 | 1 |
| 1,000 | 1 | 1 | 1 | 1 | 1 |
| 5,000 | 1 | 1 | 1 | 1 | 1 |
| 10,000 | 1 | 1 | 1 | 1 | 1 |

|  | 2 | 6 | 12 | 18 | 24 |
| --- | --- | --- | --- | --- | --- |
| 250 | 0.43 | 0.43 | 0.34 | 0.43 | 0.50 |
| 500 | 0.28 | 0.19 | 0.13 | 0.13 | 0.13 |
| 1,000 | 0.29 | 0.34 | 0.30 | 0.34 | 0.39 |
| 5,000 | 0.54 | 0.72 | 0.43 | 0.43 | 0.43 |
| 10,000 | 0.39 | 0.50 | 0.49 | 0.50 | 0.43 |

1. male (date of diagnosis, one year prior to diagnosis, date of birth)

|  | 2 | 6 | 12 | 18 | 24 |
| --- | --- | --- | --- | --- | --- |
| 250 | 0.39 | 0.63 | 0.28 | 0.28 | 0.28 |
| 500 | 0.34 | 0.72 | 0.28 | 0.41 | 0.48 |
| 1,000 | 0.29 | 0.33 | 0.26 | 0.28 | 0.28 |
| 5,000 | 0.26 | 0.14 | 0.28 | 0.54 | 0.65 |
| 10,000 | 0.34 | 0.14 | 0.28 | 0.34 | 0.54 |

|  | 2 | 6 | 12 | 18 | 24 |
| --- | --- | --- | --- | --- | --- |
| 250 | 0.22 | 0.45 | 0.22 | 0.22 | 0.22 |
| 500 | 0.22 | 0.45 | 0.22 | 0.22 | 0.22 |
| 1,000 | 0.22 | 0.22 | 0.22 | 0.22 | 0.22 |
| 5,000 | 0.32 | 0.25 | 0.65 | 0.84 | 0.85 |
| 10,000 | 0.61 | 0.22 | 0.32 | 0.53 | 0.72 |

|  | 2 | 6 | 12 | 18 | 24 |
| --- | --- | --- | --- | --- | --- |
| 250 | 0.77 | 0.77 | 0.77 | 0.77 | 0.77 |
| 500 | 0.84 | 0.84 | 0.84 | 0.84 | 0.84 |
| 1,000 | 0.84 | 0.84 | 0.84 | 0.84 | 0.84 |
| 5,000 | 0.77 | 0.77 | 0.77 | 0.77 | 0.77 |
| 10,000 | 0.77 | 0.77 | 0.77 | 0.77 | 0.77 |

1. 0–0.99 years old (date of diagnosis, one year prior to diagnosis, date of birth)

|  | 2 | 6 | 12 | 18 | 24 |
| --- | --- | --- | --- | --- | --- |
| 250 | 1 | 1 | 1 | 1 | 1 |
| 500 | 1 | 1 | 1 | 1 | 1 |
| 1,000 | 1 | 1 | 1 | 1 | 1 |
| 5,000 | 1 | 1 | 1 | 1 | 1 |
| 10,000 | 1 | 1 | 1 | 1 | 1 |

|  | 2 | 6 | 12 | 18 | 24 |
| --- | --- | --- | --- | --- | --- |
| 250 | NA | NA | NA | NA | NA |
| 500 | NA | NA | NA | NA | NA |
| 1,000 | NA | NA | NA | NA | NA |
| 5,000 | NA | NA | NA | NA | NA |
| 10,000 | NA | NA | NA | NA | NA |

|  | 2 | 6 | 12 | 18 | 24 |
| --- | --- | --- | --- | --- | --- |
| 250 | 1 | 1 | 1 | 1 | 1 |
| 500 | 1 | 1 | 1 | 1 | 1 |
| 1,000 | 1 | 1 | 1 | 1 | 1 |
| 5,000 | 1 | 1 | 1 | 1 | 1 |
| 10,000 | 1 | 1 | 1 | 1 | 1 |

1. 1–9.99 years old (date of diagnosis, one year prior to diagnosis, date of birth)

|  | 2 | 6 | 12 | 18 | 24 |
| --- | --- | --- | --- | --- | --- |
| 250 | 1 | 0.58 | 0.31 | 0.39 | 0.53 |
| 500 | 0.82 | 0.76 | 0.51 | 0.51 | 0.78 |
| 1,000 | 0.87 | 0.39 | 0.17 | 0.17 | 0.17 |
| 5,000 | 0.51 | 0.41 | 0.51 | 0.51 | 0.59 |
| 10,000 | 0.51 | 0.39 | 0.28 | 0.36 | 0.39 |

|  | 2 | 6 | 12 | 18 | 24 |
| --- | --- | --- | --- | --- | --- |
| 250 | 1 | 0.68 | 0.60 | 0.60 | 0.64 |
| 500 | 0.68 | 0.67 | 0.60 | 0.60 | 0.68 |
| 1,000 | 0.71 | 0.60 | 0.15 | 0.15 | 0.15 |
| 5,000 | 0.68 | 0.66 | 0.68 | 0.71 | 0.67 |
| 10,000 | 0.71 | 0.68 | 0.66 | 0.71 | 0.71 |

|  | 2 | 6 | 12 | 18 | 24 |
| --- | --- | --- | --- | --- | --- |
| 250 | 0.74 | 0.82 | 0.97 | 0.97 | 0.97 |
| 500 | 0.82 | 0.78 | 0.82 | 0.97 | 0.82 |
| 1,000 | 0.61 | 0.66 | 0.82 | 0.82 | 0.82 |
| 5,000 | 0.97 | 0.98 | 0.97 | 0.61 | 0.61 |
| 10,000 | 0.97 | 0.97 | 0.97 | 0.87 | 0.66 |

1. 10–17.99 years old (date of diagnosis, one year prior to diagnosis, date of birth)

|  | 2 | 6 | 12 | 18 | 24 |
| --- | --- | --- | --- | --- | --- |
| 250 | 1 | 1 | 1 | 1 | 1 |
| 500 | 1 | 1 | 1 | 1 | 1 |
| 1,000 | 1 | 1 | 1 | 1 | 1 |
| 5,000 | 1 | 1 | 1 | 1 | 1 |
| 10,000 | 1 | 1 | 1 | 1 | 1 |

|  | 2 | 6 | 12 | 18 | 24 |
| --- | --- | --- | --- | --- | --- |
| 250 | 1 | 1 | 0.99 | 1 | 1 |
| 500 | 1 | 1 | 0.14 | 0.26 | 0.40 |
| 1,000 | 1 | 0.81 | 0.40 | 0.81 | 0.73 |
| 5,000 | 1 | 1 | 1 | 1 | 1 |
| 10,000 | 1 | 1 | 1 | 1 | 1 |

|  | 2 | 6 | 12 | 18 | 24 |
| --- | --- | --- | --- | --- | --- |
| 250 | 1 | 1 | 1 | 1 | 1 |
| 500 | 1 | 1 | 1 | 1 | 1 |
| 1,000 | 1 | 1 | 1 | 1 | 1 |
| 5,000 | 1 | 1 | 1 | 1 | 1 |
| 10,000 | 1 | 1 | 1 | 1 | 1 |

1. year of diagnosis (1990-1999, 2000-2009, 2010-2019)

|  | 2 | 6 | 12 | 18 | 24 |
| --- | --- | --- | --- | --- | --- |
| 250 | 1 | 0.98 | 0.98 | 0.98 | 0.98 |
| 500 | 0.98 | 0.98 | 0.98 | 0.98 | 0.98 |
| 1,000 | 0.98 | 0.98 | 0.98 | 0.98 | 0.98 |
| 5,000 | 0.98 | 0.98 | 0.98 | 0.98 | 0.98 |
| 10,000 | 0.98 | 0.98 | 0.98 | 0.98 | 0.98 |

|  | 2 | 6 | 12 | 18 | 24 |
| --- | --- | --- | --- | --- | --- |
| 250 | 0.99 | 0.99 | 0.99 | 0.99 | 0.99 |
| 500 | 0.99 | 0.99 | 0.99 | 0.99 | 0.99 |
| 1,000 | 0.99 | 0.99 | 0.99 | 0.99 | 0.99 |
| 5,000 | 0.99 | 0.99 | 0.99 | 0.99 | 0.99 |
| 10,000 | 0.99 | 0.99 | 0.99 | 0.99 | 0.99 |

|  | 2 | 6 | 12 | 18 | 24 |
| --- | --- | --- | --- | --- | --- |
| 250 | 1 | 0.30 | 0.56 | 0.56 | 0.56 |
| 500 | 1 | 0.75 | 0.76 | 0.56 | 0.56 |
| 1,000 | 0.94 | 0.51 | 0.19 | 0.16 | 0.12 |
| 5,000 | 0.56 | 0.56 | 0.56 | 0.56 | 0.66 |
| 10,000 | 0.56 | 0.30 | 0.30 | 0.11 | 0.06 |

1. ALL (date of diagnosis, one year prior to diagnosis, date of birth)

|  | 2 | 6 | 12 | 18 | 24 |
| --- | --- | --- | --- | --- | --- |
| 250 | 1 | 0.72 | 0.40 | 0.40 | 0.40 |
| 500 | 0.95 | 0.84 | 0.50 | 0.40 | 0.40 |
| 1,000 | 0.99 | 0.40 | 0.40 | 0.40 | 0.40 |
| 5,000 | 0.43 | 0.40 | 0.40 | 0.71 | 0.72 |
| 10,000 | 0.72 | 0.40 | 0.40 | 0.40 | 0.40 |

|  | 2 | 6 | 12 | 18 | 24 |
| --- | --- | --- | --- | --- | --- |
| 250 | 1 | 0.77 | 0.32 | 0.32 | 0.49 |
| 500 | 0.58 | 0.58 | 0.32 | 0.32 | 0.58 |
| 1,000 | 0.85 | 0.51 | 0.32 | 0.32 | 0.32 |
| 5,000 | 0.68 | 0.58 | 0.58 | 0.71 | 0.58 |
| 10,000 | 0.85 | 0.58 | 0.58 | 0.58 | 0.58 |

|  | 2 | 6 | 12 | 18 | 24 |
| --- | --- | --- | --- | --- | --- |
| 250 | 0.47 | 0.80 | 0.85 | 0.90 | 0.93 |
| 500 | 0.38 | 0.38 | 0.38 | 0.38 | 0.38 |
| 1,000 | 0.38 | 0.38 | 0.38 | 0.38 | 0.38 |
| 5,000 | 0.93 | 0.93 | 0.85 | 0.38 | 0.54 |
| 10,000 | 0.85 | 0.54 | 0.68 | 0.38 | 0.38 |

1. ALL 1.5-5.99 years old (date of diagnosis, one year prior to diagnosis, date of birth)

|  | 2 | 6 | 12 | 18 | 24 |
| --- | --- | --- | --- | --- | --- |
| 250 | 1 | 0.65 | 0.36 | 0.55 | 0.59 |
| 500 | 0.65 | 0.69 | 0.65 | 0.71 | 0.83 |
| 1,000 | 0.89 | 0.44 | 0.30 | 0.30 | 0.12 |
| 5,000 | 0.85 | 0.74 | 0.74 | 0.65 | 0.65 |
| 10,000 | 0.74 | 0.65 | 0.65 | 0.65 | 0.65 |

|  | 2 | 6 | 12 | 18 | 24 |
| --- | --- | --- | --- | --- | --- |
| 250 | 1 | 0.77 | 0.48 | 0.62 | 0.77 |
| 500 | 0.48 | 0.48 | 0.48 | 0.77 | 0.77 |
| 1,000 | 0.77 | 0.48 | 0.48 | 0.48 | 0.48 |
| 5,000 | 0.95 | 0.85 | 0.85 | 0.77 | 0.72 |
| 10,000 | 0.89 | 0.77 | 0.77 | 0.85 | 0.85 |

|  | 2 | 6 | 12 | 18 | 24 |
| --- | --- | --- | --- | --- | --- |
| 250 | 0.95 | 0.97 | 0.97 | 0.97 | 0.97 |
| 500 | 0.97 | 0.97 | 0.97 | 0.97 | 0.97 |
| 1,000 | 0.97 | 0.97 | 0.97 | 0.97 | 0.97 |
| 5,000 | 0.97 | 0.97 | 0.97 | 0.97 | 0.97 |
| 10,000 | 0.97 | 0.97 | 0.97 | 0.97 | 0.97 |

1. AML (date of diagnosis, one year prior to diagnosis, date of birth)

|  | 2 | 6 | 12 | 18 | 24 |
| --- | --- | --- | --- | --- | --- |
| 250 | 1 | 0.56 | 0.56 | 0.56 | 0.56 |
| 500 | 1 | 0.56 | 0.56 | 0.56 | 0.56 |
| 1,000 | 0.56 | 0.56 | 0.56 | 0.56 | 0.56 |
| 5,000 | 0.56 | 0.56 | 0.56 | 0.56 | 0.56 |
| 10,000 | 0.56 | 0.56 | 0.56 | 0.56 | 0.56 |

|  | 2 | 6 | 12 | 18 | 24 |
| --- | --- | --- | --- | --- | --- |
| 250 | 1 | 0.68 | 0.68 | 0.72 | 0.78 |
| 500 | 1 | 0.68 | 0.68 | 0.72 | 0.78 |
| 1,000 | 0.68 | 0.68 | 0.68 | 0.68 | 0.68 |
| 5,000 | 0.77 | 0.68 | 0.68 | 0.68 | 0.85 |
| 10,000 | 0.87 | 0.72 | 0.68 | 0.68 | 0.68 |

|  | 2 | 6 | 12 | 18 | 24 |
| --- | --- | --- | --- | --- | --- |
| 250 | 1 | 1 | 1 | 1 | 1 |
| 500 | 1 | 1 | 1 | 1 | 1 |
| 1,000 | 1 | 1 | 1 | 1 | 1 |
| 5,000 | 1 | 1 | 1 | 1 | 1 |
| 10,000 | 1 | 1 | 1 | 1 | 1 |

1. one residential place (date of diagnosis, one year prior to diagnosis, date of birth)

|  | 2 | 6 | 12 | 18 | 24 |
| --- | --- | --- | --- | --- | --- |
| 250 | 1 | 0.95 | 0.38 | 0.27 | 0.41 |
| 500 | 0.95 | 0.76 | 0.27 | 0.27 | 0.44 |
| 1,000 | 0.97 | 0.95 | 0.50 | 0.44 | 0.95 |
| 5,000 | 0.97 | 0.97 | 0.95 | 0.95 | 0.95 |
| 10,000 | 1 | 0.95 | 0.95 | 0.95 | 0.95 |

|  | 2 | 6 | 12 | 18 | 24 |
| --- | --- | --- | --- | --- | --- |
| 250 | 1 | 0.95 | 0.38 | 0.27 | 0.41 |
| 500 | 0.95 | 0.76 | 0.27 | 0.27 | 0.44 |
| 1,000 | 0.97 | 0.95 | 0.50 | 0.44 | 0.95 |
| 5,000 | 0.97 | 0.97 | 0.95 | 0.95 | 0.95 |
| 10,000 | 1 | 0.95 | 0.95 | 0.95 | 0.95 |

|  | 2 | 6 | 12 | 18 | 24 |
| --- | --- | --- | --- | --- | --- |
| 250 | 0.08 | 0.20 | 0.37 | 0.52 | 0.76 |
| 500 | 0.08 | 0.17 | 0.14 | 0.37 | 0.37 |
| 1,000 | 0.37 | 0.37 | 0.37 | 0.37 | 0.44 |
| 5,000 | 0.80 | 0.89 | 0.80 | 0.80 | 0.80 |
| 10,000 | 0.80 | 0.80 | 0.80 | 0.86 | 0.82 |

*Color codes: Yellow = Suggestive of clustering, 0.05 < p < 0.1, Light grey = No clustering, Dark grey = Not analyzed.*

*Abbreviations: ALL, Acute lymphoblastic leukemia; AML, Acute myeloid leukemia; NA, Not applicable*
