# Supplementary material for: Clustering of childhood acute leukemia in Finland: a nationwide register-based study
Source: Cancer Causes Control. 2025 Apr 24;36(9):957–64. doi: 10.1007/s10552-025-01998-1 (PMC12380987; doi:10.1007/s10552-025-01998-1)
Supplement: Supplementary file 5 — Supplementary file5 (DOCX 22 KB) [file 10552_2025_1998_MOESM5_ESM.docx]

| **Table S5.** Results of the Cuzick-Edwards’ test (k=5) for leukemia (case) versus diabetes (control) based on place of residence at three residence timing categories. Smaller than expected test statistic indicates that leukemia cases are associated with a higher incidence of nearby diabetes cases. | |
| --- | --- |
| \| **At the time of diagnosis** \| **Subgroup** \| **Obs** \| **Exp** \| **Obs/Exp** \| **95%CI** \| **p-value^†^** \| \| --- \| --- \| --- \| --- \| --- \| --- \| --- \| \| **Leukemia versus Diabetes** \| All \| 681 \| 672 \| 1.01 \| 0.92–1.11 \| 0.87 \| \| Sex \| Female \| 364 \| 322 \| 1.13 \| 1.00–1.27 \| 0.72 \| \|  \| Male \| 338 \| 351 \| 0.96 \| 0.84–1.09 \| 0.85 \| \| Age, years \| 0–0.99 \| 149 \| 133 \| 1.12 \| 0.95–1.29 \| 0.85 \| \|  \| 1–9.99 \| 546 \| 560 \| 0.98 \| 0.87–1.07 \| 0.85 \| \|  \| 10–17.99 \| 113 \| 107 \| 1.06 \| 0.82–1.29 \| 0.85 \| \| Leukemia subtype, years \| ALL \| 458 \| 470 \| 0.97 \| 0.86–1.09 \| 0.99 \| \|  \| ALL, 1.5–5.99 \| 120 \| 144 \| 0.83 \| 0.63–1.04 \| 0.85 \| \|  \| AML \| 14 \| 14 \| 1.00 \| 0.33–1.68 \| 0.85 \| \| **One year prior to diagnosis** \| **Subgroup** \| **Obs** \| **Exp** \| **Obs/Exp** \| **95% CI** \| **p-value^†^** \| \| **Leukemia versus Diabetes** \| All \| 731 \| 712 \| 1.03 \| 0.94–1.12 \| 0.85 \| \| Sex \| Female \| 390 \| 340 \| 1.15 \| 1.02–1.28 \| 0.72 \| \|  \| Male \| 378 \| 372 \| 1.02 \| 0.89–1.14 \| 0.87 \| \| Age \| 0–0.99 years old \| NA \| NA \| NA \| NA \| NA \| \|  \| 1–9.99 years old \| 600 \| 593 \| 1.01 \| 0.92–1.11 \| 0.87 \| \|  \| 10–17.99 years old \| 118 \| 115 \| 1.03 \| 0.80–1.25 \| 0.87 \| \| Leukemia subtype \| ALL \| 515 \| 498 \| 1.03 \| 0.93–1.14 \| 0.85 \| \|  \| ALL (1.5–5.99 years old) \| 139 \| 152 \| 0.91 \| 0.71–1.11 \| 0.85 \| \|  \| AML \| 17 \| 15 \| 1.13 \| 0.50–1.81 \| 0.85 \| \| **At birth** \| **Subgroup** \| **Obs** \| **Exp** \| **Obs/Exp** \| **95% CI** \| **p-value^†^** \| \| **Leukemia versus Diabetes** \| All \| 743 \| 712 \| 1.04 \| 0.95–1.13 \| 0.85 \| \| Sex \| Female \| 330 \| 340 \| 0.97 \| 0.84–1.10 \| 0.85 \| \|  \| Male \| 363 \| 372 \| 0.98 \| 0.85–1.10 \| 0.85 \| \| Age \| 0–0.99 years old \| 147 \| 133 \| 1.11 \| 0.94–1.27 \| 0.85 \| \|  \| 1–9.99 years old \| 564 \| 593 \| 0.95 \| 0.85–1.05 \| 0.85 \| \|  \| 10–17.99 years old \| 121 \| 115 \| 1.05 \| 0.82–1.28 \| 0.85 \| \| Leukemia subtype \| ALL \| 486 \| 498 \| 0.98 \| 0.87–1.09 \| 0.85 \| \|  \| ALL (1.5–5.99 years old) \| 137 \| 152 \| 0.90 \| 0.70–1.10 \| 0.85 \| \|  \| AML \| 21 \| 15 \| 1.40 \| 0.77–2.08 \| 0.85 \| | |
|  |  |
| *^†^Benjamini–Hochberg adjusted p-value*  *Bold type: p-value < 0.05.*  *Abbreviations: Obs, observed; Exp, expected; CI, confidence interval; ALL, Acute lymphoblastic leukemia; AML, Acute myeloid leukemia; NA, Not applicable* |  |
