# Supplementary material for: Clustering of childhood acute leukemia in Finland: a nationwide register-based study
Source: Cancer Causes Control. 2025 Apr 24;36(9):957–64. doi: 10.1007/s10552-025-01998-1 (PMC12380987; doi:10.1007/s10552-025-01998-1)
Supplement: Supplementary file 6 — Supplementary file6 (DOCX 23 KB) [file 10552_2025_1998_MOESM6_ESM.docx]

| **Table S6.** Results of the Cuzick-Edwards’ test (k=15) for leukemia (case) versus diabetes (control) based on place of residence at three residence timing categories. Smaller than expected test statistic indicates that leukemia cases are associated with a higher incidence of nearby diabetes cases. | |
| --- | --- |
| \| **At the time of diagnosis** \| **Subgroup** \| **Obs** \| **Exp** \| **Obs/Exp** \| **95%CI** \| **p-value^†^** \| \| --- \| --- \| --- \| --- \| --- \| --- \| --- \| \| **Leukemia versus Diabetes** \| All \| 1,997 \| 2,016 \| 0.99 \| 0.94-1.05 \| 0.96 \| \| Sex \| Female \| 1,065 \| 965 \| 1.10 \| 1.02-1.64 \| 0.14 \| \|  \| Male \| 996 \| 1,052 \| 0.95 \| 0.87-1.02 \| 0.96 \| \| Age, years \| 0–0.99 \| 406 \| 400 \| 1.02 \| 0.91-1.12 \| 0.96 \| \|  \| 1–9.99 \| 1,667 \| 1,680 \| 0.99 \| 0.93-10.05 \| 0.96 \| \|  \| 10–17.99 \| 315 \| 321 \| 0.98 \| 0.84-1.12 \| 0.96 \| \| Leukemia subtype, years \| ALL \| 1,383 \| 1,410 \| 0.98 \| 0.91-1.05 \| 0.96 \| \|  \| ALL, 1.5–5.99 \| 411 \| 432 \| 0.95 \| 0.83-1.07 \| 0.96 \| \|  \| AML \| 40 \| 42 \| 0.95 \| 0.55-1.36 \| 0.96 \| \| **One year prior to diagnosis** \| **Subgroup** \| **Obs** \| **Exp** \| **Obs/Exp** \| **95% CI** \| **p-value^†^** \| \| **Leukemia versus Diabetes** \| All \| 2,142 \| 2,136 \| 1.00 \| 0.95-1.06 \| 0.96 \| \| Sex \| Female \| 1,161 \| 1,021 \| 1.14 \| **1.06-1.12** \| **0.014** \| \|  \| Male \| 1,072 \| 1,117 \| 0.96 \| 0.89-1.03 \| 0.96 \| \| Age \| 0–0.99 years old \| NA \| NA \| NA \| NA \| 0.96 \| \|  \| 1–9.99 years old \| 1,774 \| 1,779 \| 1.00 \| 0.94-1.06 \| 0.96 \| \|  \| 10–17.99 years old \| 349 \| 346 \| 1.01 \| 0.87-1.14 \| 0.96 \| \| Leukemia subtype \| ALL \| 1,496 \| 1,493 \| 1.00 \| 0.94-1.07 \| 0.96 \| \|  \| ALL (1.5–5.99 years old) \| 437 \| 457 \| 0.96 \| 0.84-1.07 \| 0.96 \| \|  \| AML \| 39 \| 44 \| 0.89 \| 0.50-1.27 \| 0.96 \| \| **At birth** \| **Subgroup** \| **Obs** \| **Exp** \| **Obs/Exp** \| **95% CI** \| **p-value^†^** \| \| **Leukemia versus Diabetes** \| All \| 2,109 \| 2,136 \| 0.99 \| 0.93-1.04 \| 0.96 \| \| Sex \| Female \| 1,019 \| 1,021 \| 1.00 \| 0.92-1.08 \| 0.96 \| \|  \| Male \| 1,096 \| 1,117 \| 0.98 \| 0.91-1.06 \| 0.96 \| \| Age \| 0–0.99 years old \| 414 \| 400 \| 1.04 \| 0.93-1.14 \| 0.96 \| \|  \| 1–9.99 years old \| 1,767 \| 1,779 \| 0.99 \| 0.93-1.05 \| 0.96 \| \|  \| 10–17.99 years old \| 337 \| 346 \| 0.97 \| 0.84-1.11 \| 0.96 \| \| Leukemia subtype \| ALL \| 1,459 \| 1,493 \| 0.98 \| 0.91-1.04 \| 0.96 \| \|  \| ALL (1.5–5.99 years old) \| 407 \| 457 \| 0.89 \| 0.77-1.01 \| 0.62 \| \|  \| AML \| 54 \| 44 \| 1.23 \| 0.84-1.61 \| 0.96 \| | |
|  |  |
| *^†^Benjamini–Hochberg adjusted p-value*  *Bold type: p-value < 0.05.*  *Abbreviations: Obs, observed; Exp, expected; CI, confidence interval; ALL, Acute lymphoblastic leukemia; AML, Acute myeloid leukemia; NA, Not applicable* |  |
